# Human altruism, evolution and moral philosophy

**DOI:** 10.1098/rsos.170441

**Published:** 2017-08-09

**Authors:** William J. FitzPatrick

**Affiliations:** Department of Philosophy, University of Rochester, Rochester, NY, USA

**Keywords:** altruism, evolution, ethical realism, debunking arguments, morality

## Abstract

This paper has two central aims. The first is to explore philosophical complications that arise when we move from (i) explaining the evolutionary origins of genetically influenced traits associated with human cooperation and altruism, to (ii) explaining present manifestations of human thought, feeling and behaviour involving cooperation and altruism. While the former need only appeal to causal factors accessible to scientific inquiry, the latter must engage also with a distinctive form of explanation, i.e. reason-giving explanation, which in turn raises important philosophical questions, the answers to which will affect the nature of the ultimate explanations of our moral beliefs and related actions. On one possibility I will explore, this explanatory project cannot avoid engaging with first-order ethical theory. The second aim is to apply lessons from these explanatory complications to the critique of ‘evolutionary debunking arguments’, which seek to debunk morality, or at least objective construals of it (i.e. moral realism), by appeal to allegedly scientific debunking explanations of our moral beliefs that would defeat our justification for them. The explanatory complications brought out in the first half raise difficulties for such debunking arguments. If we avoid begging central philosophical questions then such debunking arguments pose little threat of saddling us with moral scepticism or subjectivism, though they do pose an important challenge for those developing a moral realist view.

## 1. Introduction

Altruism and cooperation are commonly regarded as central aspects of morality. They are therefore of natural concern to moral philosophers who pose normative questions about how we should live (e.g. how much altruism is morally required of us?) and who seek to understand the status of answers to such questions (e.g. how should we understand the *meaning* of a moral claim such as ‘wealthy people are morally obligated to help the poor’, and does the meaning of such claims allow them to be literally *true* or *false*, in which case what might *ground* the truth of such claims?). At the same time, the thoughts, motivation and behaviour associated with altruism and cooperation are empirical phenomena that can be studied scientifically by evolutionary biologists, anthropologists, sociologists and psychologists. Here we might inquire into the evolutionary origins of human capacities and dispositions associated with altruism and cooperation, and more generally the origins of our ability and tendency to make (and to be motivated by) distinctively moral judgements, all of which might be spoken of broadly as inquiry into ‘the evolution of morality’ [1].

All of this raises interesting questions about how the *scientific* and *philosophical* projects might interact and bear on one another. For example, scientists inquiring into the origins of the human capacity and disposition to make moral judgements are investigating how we came to be able and disposed to make the very sorts of judgement moral philosophers are busy defending or critiquing (in first-order moral theory) or are subjecting to semantic, metaphysical and epistemic scrutiny (asking meta-ethical questions about their meaning, potential truth, grounds of truth, and so on). On one common view, this universal human capacity and disposition evolved precisely because of the fitness-enhancing effects of such belief-forming dispositions, due to their promotion of cooperative behaviours that in one way or another increased the biological fitness of ancestral humans. Human beings may have evolved the capacity and disposition to make moral judgements in the first place because of the positive coordinating effects of judgements involving moral norms (both through guiding individual behaviour through internalized norms and through public condemnation of cheating or defection, helping to enforce such norms), making for fewer ‘altruism failures’ and allowing for more successful cooperation and greater stability in larger groups, with consequent enhancement of biological fitness for participants [2]. That is an interesting claim about how we came to be able and disposed to make moral judgements of the sort also studied in various ways by moral philosophers, and it brings out the very different roles a concept such as *altruism* plays in different sorts of inquiry into morality.

Where things get especially interesting, however, is when we move from questions about the evolutionary origins of such capacities and dispositions to questions about the explanation of our current exercises of our moral capacities—the explanation of present manifestations of thought, feeling and behaviour related, for example, to cooperation and altruism. This is partly because the latter obviously reflect causal inputs stemming from *cultural* as well as biological evolution, which adds levels of complexity to the scientific causal explanation of such phenomena, bringing in disciplines such as anthropology, sociology and psychology. But there are also *philosophically* interesting complications of a quite different sort when it comes to this sphere of explanation, specifically involving our moral beliefs (e.g. our beliefs about moral goodness and badness, rightness and wrongness), and the feelings and behaviour stemming from them, which we are disposed to take very seriously as committed moral agents ourselves.

My purpose here is to illuminate these philosophical complications by exploring another dimension of explanation that arises in this context—namely, what is often called ‘reason-giving explanation’—and showing how it bears on an important philosophical debate over the relevance of evolutionary biology to questions about the status of morality. Specifically, the debate is over the ways in which evolutionary explanations pertaining to moral belief might bear on the question whether, as some of us think, there are *objective and knowable moral truths*, i.e. truths about what is good or bad, right or wrong, where these truths are not a function of our attitudes, commitments, or practices, and are, like other familiar objective truths, knowable by us—a philosophical position known as ‘moral realism’.^1^ For example, on a realist view, it might be straightforwardly true that wanton cruelty to animals is wrong, where the grounds of this truth are independent of our contingent attitudes or commitments or conventions (being rooted instead just in the badness of the animal suffering involved), and we might be in a position to know that moral fact no less than we know a variety of empirical facts. We might then ask: do the explanatory implications of evolution cast doubt on such a realist understanding of morality?

To put my cards on the table, my own philosophical view is indeed just such a realist one. I will not, however, be concerned here with trying to show that such a view is in fact correct. Instead, my aim is just to show how this realist possibility, which is one of the plausible philosophical candidates for understanding the status of morality, complicates explanatory projects that target our moral beliefs and related feelings and behaviours, even if we grant the evolutionary explanatory claims about the origins of certain underlying human psychological capacities and dispositions. In particular, I will raise difficulties for what have come to be known as ‘evolutionary debunking arguments’ against moral realism. Again, my aim will not be to show that the kinds of debunking explanatory stories (about why we have the moral beliefs we do) involved in such arguments could not turn out to be true, but only to show that they cannot be expected to be compelling to those of us who are independently drawn to moral realism for philosophical reasons, even granting all the scientific data. What we have in this debate are competing models of explanation for at least some of our moral beliefs, and which of them is ultimately correct will turn on deep and contentious philosophical questions, not just on scientifically tractable ones, or so I shall argue. So while various *debunking explanatory stories*, evolutionary or otherwise, could ultimately turn out to be the correct accounts of why we hold the moral beliefs we do, the science itself does not underwrite generally compelling *debunking arguments* that can be used to dislodge a philosophical position such as moral realism, which remains a viable possibility. This is relevant not only to philosophers but equally to scientists, some of whom may be attracted to moral realism in their capacity as reflective moral agents.

## 2. Scientific explanation and philosophical complications

When an ant performs a biologically ‘altruistic’ component of its job in the colony we look simply for *causes*—both remotely in evolutionary influences behind the shaping of the disposition, and proximately in causal factors leading to the token behaviour. The same will be true even in cases of behaviour involving a component of contingent ‘cultural’ innovations within a group, as in the explanation of certain cases of birdsong: though we cite ‘cultural’ developments in the recent past as part of the explanation, we are still dealing simply with ordinary causal explanations of the empirical phenomena. But when it comes to human thought and action, such as your deliberating and choosing to aid a stranger in distress, things are more complicated.

The particular complication I wish to highlight lies in the fact that in addition to familiar *scientific causal explanations* for how various empirical phenomena have come about, there is in the case of human beliefs and actions (and desires and feelings) also an importantly different form of explanation in play: namely, *reason-giving explanation* offered (in the first instance) by the objects of study themselves for at least many beliefs, actions and feelings. For we arrive at many of our beliefs, actions and feelings by way of judging that we have *good reasons* for them such that they are *justified*; and when we are asked why we believe what we do, or act or feel as we do, we offer *our reasons* for these things as both justification and explanation.

Consider first belief. In explaining why we believe what we do, we typically offer what we take to be *good reasons* for holding the belief in question, i.e. considerations that justify the belief by supporting the truth of its content. For example, to take an empirical belief: you might explain why you believe in anthropogenic climate change by citing considerations you judge to be good reasons for believing the content in question, i.e. considerations you take to be evidence that the proposition that anthropogenic climate change is occurring is in fact true. Or to take a mathematical belief: you will presumably explain why you believe that there are infinitely many prime numbers by citing something like Euclid's famous proof, which you (correctly) take to show that there *are* infinitely many primes, thus constituting *good reason* to believe the content of this mathematical proposition to be *true*. This is obviously a very different situation from your having merely been pushed by extraneous causal factors to harbour such a belief, as in the case where you believe something because a hypnotist has caused you to have the belief.

Similar points can be made with respect to actions and feelings. You might explain your altruistic act toward a stranger in dire need by citing considerations you judge to be good reasons for so acting, i.e. considerations you take to show that so acting is in fact practically justified or good to do. You might similarly offer reasons for your feeling angry over someone else's action that, let us suppose, caused this person to be in such need in the first place. Such cases involve explaining ourselves by offering purportedly *justifying reasons*—considerations we took to justify our belief, action or feeling—within a space of reasoned discourse that invites demands for such justifying reasons and allows for critical challenges to those that are offered (i.e. asking whether the reasons offered *are really good reasons* for so believing, acting or feeling).

This sort of explanatory discourse obviously contrasts with the sort of explanation that might be given for ant behaviour, and because it raises normative questions about purported justifications for belief, action or feeling, it takes us into new and philosophically interesting territory. We might ask simply what caused an ant to be aware of the location of a food source, but it will not be that simple when it comes to your beliefs about prime numbers or climate change. Instead of starting by asking what *caused* you to believe such things, as we might ask what caused you to develop a headache or to twitch just now, the appropriate place to begin in explaining why you believe what you do is to ask you your *reasons* for believing such things to be true. For as a rational agent you arrive at such beliefs not just passively, though susceptibility to causal pushes and pulls, but *through judging certain considerations to be good reasons for believing certain propositional contents to be true*. In explaining your belief, we thus start by finding out *your reasons*—the considerations you took to be good epistemic reasons for so believing.

Now once we engage in this form of explanation we will be drawn, in filling out the explanatory project more deeply, into the critical dimensions of talk of reasons, where questions arise about the quality of the justifications offered, which is assessed by assessing the quality of the reasons cited. We may judge, for example, that your reasons for your belief about prime numbers or about climate change are excellent ones, genuinely supporting those beliefs (because the proof is mathematically sound, and the climate data are strong). In other cases we may instead reject the truth of the considerations offered as reasons (e.g. if someone cites Biblical inerrancy as his reason for denying evolution), or dispute whether the considerations cited genuinely support a certain belief (e.g. if someone cites the fact that her home town had a colder than average winter as her reason for denying global warming). The point, however, is that these issues will make a difference to our ultimate understanding of why the beliefs in question are held: in one case our explanation will cite your *competent recognition of good reasons* for believing something to be true, while in another case it will have to cite causes of someone's erroneously taking some consideration to be a good reason to believe what they do. This sort of engagement with reason-giving explanation and assessment therefore ultimately takes us from the position of observers dealing exclusively with causes as such, to participants in first-order reasoning about such things as mathematics or climate science. In assessing the quality of your reasons for your belief about prime numbers, for example, which is part of achieving a deep understanding of why you ultimately believe what you do, we have to assess the soundness of the mathematical proof you cite in support of it, and this takes us into the realm of first-order mathematical reasoning; we are not dealing simply with causal factors in the way we are when explaining the aetiology of your headache.

Let us now consider the case of action and of beliefs that are specifically moral in nature, returning to the example of your helping the stranger. Suppose you gave someone money. Typically this will again be something for which you have reasons to offer, i.e. considerations you took to justify so acting, telling in favour of it, which motivated you so to act and which you will cite in defence of your action if asked why you so acted. That is, your action arose through your normative judgements about reasons, such as your thought that this person's need of money for food for herself and her children is a *good reason* for you to give her some, which you take to justify this action as a good thing to do. So as before, rather than starting by seeking ordinary causal factors to explain your action, as we would do in explaining your twitching or developing a headache, we need to start by inquiring into your reasons for so acting—the considerations you took to be good reasons for giving the money. And similarly with your related moral belief, let us suppose, that you had a moral obligation to help. Indeed, your (epistemic) reasons for holding this moral belief will be largely the same as your (practical) reasons for helping, e.g. the fact that this family is hungry and in need of money to buy food, and that you can easily spare this money without major burden or sacrifice. So in explaining why you had that moral belief we should again start there, with your reasons for it—the considerations you judged to constitute good reasons for thinking it to be true that you had such an obligation.

Now the next question, as before, will be why you made *these* normative judgements about these considerations being good (epistemic) reasons for thinking you had a moral obligation to help, and good (practical) reasons for helping. And here we run headlong into a deep and contentious philosophical issue. For philosophers are divided on whether or not there is ultimately any such thing as genuinely good or bad reasons in the sphere of ethics. Some doubt that there can be any facts at all about good or bad reasons for doing anything, or for believing anything that involves ethical concepts (such as that it's right to help a stranger in serious need when one can easily do so), since they doubt that there is any role for truth (as it is typically understood) in the ethical sphere. Such a sceptic might still *begin* his search for explaining your moral belief and altruistic action by looking to the justificatory reasons you cite in defence of them. But he will think that these normative thoughts of yours—e.g. that the fact that this person is in dire need and that you can easily help is good reason to believe that you have a moral obligation to do so, and a good reason for so acting—have nothing to do with your having competently *recognized* any normative *truth* here about good epistemic or practical reasons. That is, he will treat this case very differently from the mathematical case, where we presumably do allow that your thoughts about Euclid's proof involve your competently recognizing the (epistemically) normative truth that, being sound, the proof does indeed provide good reason for believing the proposition about prime numbers, showing it to be true.

For someone who is thus philosophically sceptical about any parallel truths in the ethical sphere, when it comes to explaining your judgements about good reasons for thinking it right to help others and for actually helping them, there will be nothing to look to other than *merely causal factors that operate independently of any relation to the truth of the content* of your judgements (there being no such thing as truth here, on the sceptical supposition). Unlike with the mathematical case, the sceptic will not be led to engage in first-order ethical inquiry as part of seeking to understand more deeply why you believe what you do about practical reasons, and so why you then act as you do on their basis. He will instead just look for *causes* of your practical judgements and actions in evolutionary influences and in cultural causal factors of the sort available to sociology and psychology, this being all there is to the story.

As noted earlier, however, that is only one philosophical possibility. Another possibility is moral realism, and more generally realism about truths concerning moral obligations, good reasons for believing certain moral propositions to be true, and good reasons for acting in certain ways. And if this realist view turns out to be correct, then the situation with respect to explaining moral beliefs and actions will be complicated in just the ways we have considered in connection with mathematical or empirical beliefs. Once we are told your reasons for holding a certain moral belief or for performing a certain action, there will be further questions about *why* you *took* those considerations to be good reasons for so believing or acting—questions that (on this realist approach) do not call simply for answers in terms of scientifically accessible causes, as the sceptic above claims, but instead require assessment of the quality of the reasons offered. We will need to find out whether your believing and acting as you do is the result of your *ethically competent recognition of genuinely good reasons* (parallel to your mathematically competent recognition of good reasons for believing that there are infinitely many primes), or whether it is instead just the result of extraneous causes (as in the case, we may suppose, where someone erroneously takes the fact that someone is a stranger to be a good reason to believe she has *no* moral obligation to help). This is still in the service of explaining your beliefs and actions, but the point is that it again takes us from the position of outside observers dealing just with scientifically accessible causes (as when explaining ant perception and behaviour, or the onset of your headache) to that of participants in *first-order ethical reasoning*, in assessing the quality of the practical reasons you cite, as part of seeking a deep understanding of your action.

What we have here, then, are two very different but still open philosophical possibilities, leading to two quite different models for what is involved in adequately explaining current human moral beliefs, feelings and actions. Both models are consistent with plausible evolutionary explanations of genetically based components of evolved human psychological and behavioural capacities and dispositions. The realist model simply goes on to posit two further things. First, it claims that although our moral beliefs and related actions obviously draw on such evolved capacities and reflect evolved dispositions to *some* extent, they are also informed and shaped by critical reflection and normative judgement emerging from cognitive and emotional faculties that have been intelligently developed and trained in rich cultural contexts within traditions of moral inquiry. Second, it must suppose that by engaging in such inquiry into first-order questions about what makes for good or bad reasons for acting or for believing various moral propositions, we are able at least sometimes to recognize truths about good reasons for belief or action, just as we do in mathematical inquiry with respect to good or bad reasons for believing propositions about numbers, and (we hope) in philosophical inquiry about matters such as the present topic.

I will return to this below. The point to emphasize here is the openness of the two philosophical possibilities and models I have sketched, neither of which is either established or refuted by the sciences themselves. In particular, it should not be assumed that what I've called the sceptical position is somehow the natural default. It is a possibility: it could be that your belief that slavery is wrong, for example, has nothing to do with its actually being wrong and with your recognizing that fact by grasping the reasons why it is wrong, as in the parallel case of your beliefs about prime numbers or climate change; perhaps there are no such facts about wrongness or about certain features of slavery (which you cite as your reasons for believing it to be wrong) being genuinely wrong-making and so being good reasons for believing that slavery is wrong. But it could equally be the case instead, as the realist model allows, that you believe that slavery is wrong because it *is* wrong and you've recognized that fact by competently recognizing good reasons for thinking it to be wrong (parallel to the story about your mathematical belief). These are, again, open philosophical questions and a matter of ongoing debate.

What this means, then, is that the task of explaining current moral beliefs, feelings and actions cannot simply be assumed to fit neatly and exhaustively into a scientific causal explanatory framework. It will indeed do so *if* the sceptical position is correct, since there will then be at bottom nothing but scientifically accessible causal factors to explain these things: though people cite reasons for their moral beliefs and actions, their taking these considerations to be good reasons will itself just be something that can be fully explained simply by appeal to empirical causal factors without having to engage in first-order ethical inquiry about which considerations ‘really are’ good reasons. But if the realist position is correct, the explanatory project will be more complicated in the ways we have seen. It may be, for example, that while your altruistic action in helping the needy stranger you encountered obviously drew upon evolved psychological capacities and may have been influenced *partly* by evolved psychological dispositions, it may also have been crucially motivated by your *recognition* that you in fact had *good reason* to help, based on your morally competent recognition that people's well-being matters and their needs provide others with good (epistemic) reasons for believing it is right to help them and good (practical) reasons for helping them.

If that is how it turns out to be, then your behaviour cannot adequately be explained simply by appeal to scientific causal explanations involving evolutionary biology, sociology, psychology, and so on—any more than we could adequately explain your mathematical beliefs in this way. Just as it will be crucial to the proper explanation of your belief about prime numbers to cite your competent *recognition* of *good reasons* for believing its content to be true (in recognizing the soundness of the proof that supports it), so too it would be crucial to the proper explanation of your altruistic behaviour toward the stranger in need to cite your competent *recognition* of *good reasons* for believing it to be true that it was right to help her, and for actually helping her. Such explanations would bottom out in ethically and philosophically implicated claims (as the explanation of your mathematical belief bottoms out in a mathematically implicated claim about the soundness of Euclid's proof, and your competent recognition of it) rather than in the sorts of causal factors that are the focus of scientific explanations.

Let me stress again that I am not here trying to show that the realist model is in fact correct. But its philosophical possibility shows that the explanation of our moral beliefs, feelings and actions may well require detours through territories outside of scientific causal inquiry, in ways that may not have been obvious. And for those of us attracted to the realist model for explaining at least some of our moral beliefs, such as the belief that slavery is wrong, or some of our behaviour, such as principled altruistic action, there is no escaping these broader inquiries in seeking a fully adequate explanation of human moral belief, feeling and action.^2^

## 3. General evolutionary debunking arguments

The above reflections highlight philosophical possibilities that complicate any attempt to move straightforwardly from evolutionary explorations of the origins of genetically based capacities and dispositions—or even from such explorations *combined* with further scientific inquiries into sociological and psychological causal influences in cultural contexts—to explanations of current moral thought, feeling and behaviour. If there is such a thing as truth and good epistemic and practical reasons in the ethical sphere, and if our recognition of normative facts about these things has played a role in our coming to believe and to act as we do (at least in some cases), then a purely empirical approach to explaining our beliefs and resultant behaviour, appealing only to scientifically accessible causal factors, will be as incomplete and misleading as such an approach would be to explaining our competent mathematical beliefs.

This point raises difficulties for increasingly popular claims that scientific work, particularly in evolutionary theory, can be used to do philosophical ‘debunking’ work in ethics. Some have claimed that appeals to evolutionary influences on human psychology can debunk morality altogether [1], or at least realist views of it [9]. Others have argued more specifically for the evolutionary debunking of certain particular ethical beliefs, such as belief in ‘partialist’ moral systems that recognize special moral duties to kin and prerogatives to favour oneself and friends and family over strangers [10]. Let me thus turn to such ‘debunking arguments’ and try to show how I believe they overreach, given the open philosophical possibilities highlighted above.

Evolutionary debunking arguments typically appeal to scientific causal explanations of our ethical beliefs either (i) to support unqualified ethical scepticism, by undermining our justification for our ethical beliefs [1], or at least (ii) to discredit ethical realism—the view, again, that there are real ethical properties and facts that are not merely functions of our ethical attitudes, commitments or practices—by showing that our justification for our ethical beliefs would be undermined by such explanations if realism were true regarding the nature of ethical properties and facts [9]. (I'll return in the next section to the more selective use of debunking arguments by de Lazari-Radek & Singer [10]). At the root of such arguments is a recognition of a fundamental disconnect between (i) *evolutionary forces*, which are assumed to have exerted considerable influence over the development of human ethical belief-forming capacities and dispositions, and (ii) the *ethical properties and facts* posited by ethical realists.

Evolution did not design human ethical belief-forming capacities and dispositions to track ethical properties and facts as such, at least not on any construal of such properties and facts that would be ethically plausible and of interest to ethical realists. Instead, natural selection would have honed ethical belief-forming capacities and dispositions (like any other traits under genetic control) through incremental modifications that were selected for simply because of their tendency ultimately to increase the biological fitness of the organisms having these modifications. Simply put, natural selection would have rewarded the capacity and disposition to form *whatever* ethical beliefs led to behaviours that caused Pleistocene hunger-gatherers to leave more offspring than peers with variant beliefs and behaviours. And importantly, the *truth* of these ethical beliefs—in the realist's sense of their accurately representing or tracking independent ethical facts (if such facts exist)—was irrelevant to that process, unlike in the case of perceptual beliefs, for example.

In the case of perceptual beliefs, the rough accuracy of the beliefs clearly mattered to the enhancement of fitness: it was precisely by giving our ancestors roughly accurate representations of their environment that the relevant perceptual beliefs contributed to their reproductive success. By contrast, in the case of ethical beliefs (and similarly religious beliefs) the *truth* of the *content* of the belief is not what mattered to enhancing fitness. Suppose beliefs in the ethical permissibility of killing step children or exploiting outsiders or philandering are false. Still, as long as such beliefs led to behaviours that increased reproductive output (as they well may have in these cases) they would play a role in the selective shaping of ancestral ethical belief-forming dispositions. Similarly with false religious beliefs: natural selection will have favoured a tendency to form such beliefs as long as they led to behaviours that increased reproductive success—as by promoting greater cooperation and adherence to social rules, out of fear of divine sanction from an ever watchful god. So the point is that natural selection did not shape our faculties to track ethical facts, even if they exist, any more than it shaped them to track theological facts: it instead shaped them to track facts relevant to competitive gene propagation in ancestral environments. And while there may be some partial overlap between the two sets of facts, there will also be plenty of divergence, which again is no surprise given that ethical properties and facts are not understood on any plausible philosophical view to have generally to do with competitive gene propagation among Pleistocene hunter-gatherers.

Now this is commonly thought by debunkers to raise serious problems for the epistemic justification of our ethical beliefs (and hence also for the possibility of ethical knowledge), at least under a realist conception of what it would be for such beliefs to be true, i.e. that it would involve their accurately representing objective ethical facts [1,9]. For given that our ethical belief-forming dispositions would not have been shaped by natural selection to track ethical facts, having been shaped only by ‘morally blind’ causal forces, it would be a *sheer coincidence* if they happened to do so reliably [9]. Unlike with our perceptual belief-forming dispositions, which were plausibly shaped to be largely reliable (this being how they enhanced ancestral fitness), our ethical belief-forming dispositions would not tend toward any parallel reliability with respect to tracking realist ethical facts. And since we have no reason to suppose that there has been some wild coincidence such that our ethical beliefs just happen to line up with these ethical facts (if there are such facts), we lose any justification for our ethical beliefs once we become aware of all of this; that is, learning of the extraneous causation of our ethical beliefs—by forces indifferent to the truth of the content of those beliefs—serves as a defeater for our epistemic justification for those beliefs. And we can likewise hardly make claims to ethical *knowledge* if it is only at best a *happy accident* that our ethical beliefs line up with the ethical facts.

The situation would be much like one imagined by Richard Joyce [1], where you find out that your belief that Napoleon lost the Battle of Waterloo is entirely the product of a pill someone slipped you, which causes such a belief to be formed independently of any historical facts; there is also a pill that causes the belief that he won, and you just happened to have been given the ‘lost’ pill through a lottery. Surely this should undermine your confidence in your belief that Napoleon lost the Battle of Waterloo: it would be a sheer coincidence if your belief happened to match the historical facts, which played no role in the aetiology of your belief. But the same holds for our justification with respect to ethical beliefs if they're really just products of evolutionary and cultural ‘Napoleon pills’, which is in essence the debunker's claim. And even apart from the point about justification, realists can hardly make a convincing claim to ethical *knowledge* and *understanding* if it turns out that any accuracy of our ethical beliefs ultimately rests on a lucky fluke in this way—that our believing what we do has nothing to do with the ethical facts being what they are. In this way, many imagine that appeals to evolutionary history can be used to ‘debunk’ our ethical beliefs or claims to ethical knowledge, at least on realist assumptions about what it would be for ethical beliefs to be true (i.e. to accurately represent real ethical facts).^3^

The problem, however, is that none of this actually follows from the plausible points made about how evolution would have shaped our ethical belief-forming dispositions. Suppose it is true that our basic capacities for moral judgement and motivation evolved for reasons having nothing to do with ethical facts, arising instead from Darwinian social selection pressures operating on ancestral humans [2]. All that follows is that we shouldn't expect *natural selection* to have *made* these capacities reliable for us, pre-tuned to track ethical facts, as our perceptual capacities come pre-tuned to track facts about our environment. It doesn't follow that *we* cannot now *develop* and *train* these evolved capacities in cultural contexts in such a way that we can now exercise them reliably to track ethical facts and arrive at justified ethical beliefs and even knowledge.

Insofar as natural selection gave us specific dispositions to exercise our capacities in certain ways, making certain kinds of judgement, we cannot expect those dispositions to be reliable or truth-tracking. But this does not preclude *our* having developed other, more reliable dispositions through independent uses and training of our evolved faculties in cultural contexts involving traditions of ethical inquiry, as noted earlier. And these dispositions may well be employed to override certain evolutionarily given ones, as we learn to suppress instinctive patterns of ethical thought we come to regard as irrational or unfounded; they can also be employed to provide independent reinforcement for other innate dispositions in cases where we judge that they happen to be accurate, as in dispositions to care for our children, which incline us helpfully toward the judgement that we should.

There is nothing fanciful in this idea that we have taken faculties that evolved for one reason and developed and trained them in cultural contexts to yield new dispositions that can be reliable for very different purposes. We have plainly done this in other domains of inquiry—such as quantum physics, algebraic topology, or for that matter philosophy itself—whose target facts were equally irrelevant to the Pleistocene evolutionary shaping of the basic cognitive capacities we draw upon in discovering those facts today. Indeed, that is just what we're doing in this very philosophical debate about evolution and epistemology, which natural selection certainly didn't ‘design’ Pleistocene hunter-gatherers to do accurately either.^4^ The point, then, is that it is so far a wide-open possibility that we have done something similar in *ethical* reflection and judgement as well, even on a realist construal of ethical facts.

In order to raise a special problem for ethical realism the debunker would therefore have to close off that possibility by undermining the suggestion that, just as in other domains of inquiry, we have similarly developed and trained the various capacities bequeathed by natural selection in such a way as to conduct largely successful inquiry into ethical facts, giving ourselves fairly reliable ethical belief-forming dispositions. Perhaps the debunker's claim here will be that while in other domains we're simply *extending* forms of reasoning we did evolve to do accurately, in ethical reflection and judgement we are engaged in something entirely different, with no plausible basis for an extension to something reliable for other purposes. But this thought relies on a caricature of our ethical capacities as little more than evolved modules or ‘computational mechanisms’ for producing fitness-enhancing judgements and motivation. Even if there are some such modules, our ethical capacities consist also in our more general reasoning abilities, including the broad capacity to employ evaluative and normative concepts in relation to relevant standards in a variety of contexts, and to reason critically about such claims. And this is something we *were* plausibly shaped to be able to do well.

Our ancestors presumably needed to engage in accurate evaluative thinking about such things as good and bad dwelling places, or hunting partners, or fighters, or food, and related normative thinking, as in the judgement that one ought not to eat the little brown mushrooms, given the aim of not dying. There would therefore have been selective pressure in favour of accurate evaluative and normative thinking in everyday practical spheres. This capability could then be combined with a capacity for moral judgement, allowing human intelligence to be applied in developed cultural contexts to extend cruder frameworks so that we now think evaluatively in relation to standards pertaining to what it is generally to live well and to be a good human being [6]. If we have been able to take mental capacities that evolved because they promoted reproductive success in hunter-gatherers and now develop and deploy them to do ten-dimensional mathematics, then it's hardly a greater stretch to suppose that we've similarly been able to develop and extend our evolved capacities so as to be able to figure out, with input from ongoing personal and social experience and improvements in background knowledge, that racist voting restrictions are unfair and wrong, for example, or that girls shouldn't be attacked for pursuing an education (assuming, with the realist, that there are such facts).

Now let me again emphasize that it is not my purpose here to try to show that such a realist picture is in fact correct and that we have indeed been able to arrive at such moral knowledge, though I believe we have. (There are many familiar sceptical worries one could raise about the reliability of reasoning when applied to the ethical sphere, which would need to be addressed in a full philosophical defence of realist moral knowledge.) Instead, the point so far is just that this sort of picture is in any case compatible with what the sciences themselves tell us: it is not somehow precluded by the scientific data. Let us grant that evolutionary forces have plausibly had *some* influence on *some* of our ethical beliefs, and that to the extent they have done so we cannot *on that basis* expect those beliefs to be accurate, since natural selection wasn't in the business of shaping our ethical belief-forming dispositions to be truth-tracking. Similarly, let us grant that there have been many other causal factors of the sort cited by sociologists or economists, say, which have to some extent steered ethical beliefs while being equally blind to the realist's ethical facts. For all that, it remains an open philosophical possibility that there *are* such realist ethical facts and that we have come to know at least some of them through developing and training our moral faculties in such a way that, with input from ongoing experience and growing empirical knowledge, we are able to exercise those faculties in ways that enable us, in moral reflection and deliberation, to recognize *good reasons* for holding certain moral beliefs or for acting in certain ways, as described in the previous section. Moreover, this possibility is not merely an idle one. It is in fact quite plausible when we consider morality not just ‘from the outside’ but from our perspective as engaged moral agents.

Consider a simple example. After hearing of the Taliban's attempted murder of Malala Yousafzai on her school bus for advocating educational rights for girls, we might find ourselves with the strong belief that such behaviour is morally heinous, and more generally that it is seriously wrong for anyone, from any culture, to deprive girls of educational and career opportunities simply because they are girls. Now some will no doubt claim (following the sceptic described earlier) that a fully adequate and exhaustive explanation of our belief can be provided simply in terms of sociological, psychological, or historical causes operating in ways that are insensitive to any real ethical properties (such as wrongness) or ethical facts. But no one who, as a result of reflection on core ethical beliefs as a committed moral agent, has realist leanings and is serious about this ethical belief concerning the badness of such violent, sexist practices should rush to accept such a claim. We will not regard our ethical reactions here as mere empirical phenomena to be explained using scientific tools, on the model of headaches or mental states resulting from mere cultural conditioning (as with fashion-related desires); nor will we concede that explanations appealing only to scientifically accessible causes are the ‘best explanations’, and that realist appeals to ethical properties or facts are a needless extravagance [2,12].

Instead, we will take the ethical belief in question to result from our recognition of good reasons for taking it to be true, which will thus be crucial to the proper explanation of it—even if this is less parsimonious than scientific explanations [7]. If asked why we believe that these Talibanic practices are morally wrong we will cite reasons that we take to support the truth of the belief, not merely psychological or sociological *causes* for it that operate independently of such reasons. Such practices, we will emphasize, are unjust, cruel, demeaning and sexist, violating human rights and dignity by depriving girls of central human capabilities and goods, based on arbitrary considerations. We cite these considerations, against our background view of the standards of moral excellence for human beings and action, as *wrong-making features* of these practices, which thus constitute *good reasons* for believing them to be wrong. And from a realist perspective, what is happening here is that we are competently recognizing the wrong-makingness of these factors, and this is then precisely what leads to our ethical judgement: we believe the practices in question to be wrong *because* they *are* wrong and, being morally competent, we've recognized this evaluative fact by grasping the reasons why the practices are in fact wrong, as such. As with the mathematical example, all of this is then crucial to the proper explanation of our belief.

Now let us return to debunking arguments. They are meant to be arguments against moral realism, and so do not simply presuppose that there are no objective moral truths, which would be obviously question-begging. Instead, they try to show that even if there were such truths—even if it were objectively true, for example, that it is wrong to deprive girls of educational opportunities—we could not have knowledge of such things, since our moral beliefs would not be suitably related to such truths, having been produced in us by evolutionary and other ‘morally blind’ causal factors (akin to Joyce's ‘belief pill’), undermining our justification for them. The problem with such a line of argument, however, is that if we start out taking moral realism seriously and we do posit such truths, then we need never in the first place accept the debunker's premise that our moral beliefs are, across the board, nothing but causal upshots of ‘morally blind’ forces. If, as we start out supposing, there are objective moral truths, then there is a plausible alternative model for how we have arrived at many of our moral beliefs that would not make it a mere happy accident that they line up with these truths. That is, we may have arrived at moral beliefs such as the one described above through intelligent and informed exercises of our culturally developed moral faculties such that we have come to believe what we do through recognizing good reasons for believing the contents in question to be true. The sciences themselves do not show that this is not happening, and insofar as it remains an open and plausible possibility, those of us attracted to realism can reasonably find it more plausible than the debunker's claim about the epistemically undermining aetiology of our moral beliefs. The debunking argument thus fails to get a grip on realists in the sense of providing non-question-begging leverage to dislodge their position that there are knowable objective moral truths (though of course this doesn't give realists the upper hand either—it is a merely defensive point against such debunking arguments).

This is not to deny that some debunking explanations are true of some of our beliefs. Even if there *are* truths in the realm of ethics, sometimes we are deceived in thinking that we believe or do things for the reasons we cite in their defence [13]. And even where we are not so deceived, sometimes our judgements about good reasons will be false, in which case they obviously do not result from recognition of the relevant truths about these things, and will instead be explained at least partly in terms of extraneous causal factors—such as biases, cultural distortions, wishful thinking, and so on. Even moral realists will have recourse to such forms of explanation in accounting for why someone holds certain moral beliefs that happen to be false, such as the belief that interracial marriage is wrong. The real issue is whether there is an argument, specifically from what we know of *evolution*, to compel all of us—even those of us who start off, for philosophical reasons, as moral realists—to accept that debunking explanations are the best explanations of our moral beliefs across the board, and that our moral beliefs are therefore unjustified and so could never constitute knowledge of objective moral truths. This is what I am denying.

To be sure, *if* the kinds of explanations debunkers offer were in fact the exclusive and exhaustive explanation of our ethical beliefs quite generally, then we would indeed have a problem. But merely being able *to tell a debunking story* does not amount to showing that it is in fact the correct and exhaustive explanation of our ethical beliefs. Maybe it is, maybe it isn't. And until the alternative philosophical model I've sketched is somehow eliminated as a contender, we don't have an evolutionary *debunking* of ethics or of moral realism—at least not of a sort that might be expected to move anyone who starts out sympathetic to realism (rather than preaching to the choir of non-realists). What the arguments do plausibly show is that *to whatever extent* our current beliefs, feelings and actions have been shaped by evolutionary influences *and not* by any independent exercise of developed ethical competence of the sort I have described, we cannot expect them to be reliable. But that does not debunk ethics. Those with debunking ambitions can go on to tell a story according to which *all* our ethical beliefs are indeed *entirely* the result of such evolutionary and other equally ‘morally blind’ causal factors, but nothing in the sciences themselves compels us to accept it given the plausible alternative that remains on the table. Some will, of course, accept the debunking explanation, but this will have to be partly for *philosophical* reasons, requiring substantive and developed philosophical arguments in metaphysics, epistemology and moral philosophy, aimed at ruling out the alternative I have described; it won't come from the sciences themselves (at least insofar as the sciences are conceived, as they typically are, as empirical disciplines that do not themselves engage in philosophical inquiry into matters such as the possibility and nature of moral truth). For those of us who instead embrace some form of realist model, the ‘debunking arguments’ do not present us with a compelling debunking, though they do still present a valuable challenge to realists to develop a satisfactory moral epistemology in light of our status as evolved creatures, which needs to be taken seriously [3–8].

## 4. Selective evolutionary debunking arguments

Katarzyna de Lazari-Radek and Peter Singer [10] have taken a mixed approach to evolutionary debunking arguments. On the one hand, they reject the claim that such arguments can be used to debunk their own belief in *impartial utilitarianism*, i.e. Sidgwick's Universal Benevolence. On the other hand, they welcome such arguments against beliefs in opposing moral theories, targeting not only extreme egoism but also any moral view that, like ‘the morality of common sense’ (as Sidgwick calls it), incorporates some element of partiality, as by (i) recognizing special duties to family and friends even where this will not maximize total utility as impartially conceived, or (ii) granting prerogatives to favour oneself and one's family and friends to some degree over strangers in making decisions regarding aid, for example. This mixed strategy, however, is unstable in both directions: if the weapons Singer and de Lazari-Radek deploy against their opponents worked, they could equally be employed without much difficulty to undermine their own beliefs; and if the considerations they raise in defence of their own view succeed in protecting it against evolutionary debunking, then the same considerations can equally be used to shield the sort of commonsense morality they attack, for example.

I have argued that the mere fact that someone can tell a debunking evolutionary story that would, *if true*, cause problems for some or all ethical beliefs does not by itself debunk those beliefs: our justification for those beliefs would be defeated only if we actually had compelling reason to think that the debunking explanation *is in fact the true and exhaustive explanation* of those beliefs, showing them to be the result of nothing but extraneous causal factors operating insensitively to the truth of the content of the beliefs—as with the Napoleon belief pill. By contrast, Singer and de Lazari-Radek, in their enthusiasm to make use of evolutionary debunking against their opponents, suggest that the mere existence of a possible evolutionary explanation for certain ethical intuitions or beliefs (specifically those involving some degree of partiality) casts doubt on their reliability.

It is, of course, easy enough to imagine evolutionary explanations for why people might think they had special duties to family and friends, or could legitimately focus more altruism on their own children than on perfect strangers, even where more impartially conceived good might come from aiding strangers. And the alleged threat from debunkers is the possibility that despite the fact that these ethical beliefs have been mediated by cultural developments and refined through reasoning, all of this just traces back to ‘contaminated starting points’ with no claim to ethical reliability; it all goes back to an evolved disposition to favour oneself and one's kin over others, which has trickled down to our current commonsense ethical beliefs with *no additional input* from recognition of *good reasons* for such special forms of concern, through ethical experience, training and reflection. But again, this so far remains nothing but a sceptical possibility. Singer and de Lazari-Radek, however, are anxious to give it serious weight in discrediting such views, yet are not worried about problems for their own view because they are confident that no such evolutionary story can be told against their own belief in impartial utilitarianism.

This is playing with fire. It is, in fact, easy to come up with debunking evolutionary stories of just the same kind against their view. For example, perhaps instead of (or in addition to) kin selection operating in our distant past to give us targeted kin altruism, natural selection more simply and economically gave rise to a relatively *indiscriminate* sympathy and tendency toward altruism, among other psychological dispositions (including competing egoistic ones). In ancestral environments involving mostly small, in-group interactions, this altruistic disposition may well have contributed positively on the whole to each individual's fitness due to the benefits for each of such in-group cooperation, despite some ‘misfirings’ and ‘wasteful’ altruism when directed toward non-cooperators or outsiders [14]. A debunking explanation of current beliefs in impartial utilitarianism might thus see them as nothing but an extension of such ‘misfiring’ of that evolved sympathy and altruism, through cultural developments and refinements. Reasoning may be involved in eliminating various forms of parochialism, driving Singer's ‘expanding circle’ of moral concern (even to non-human animals), but according to the debunking story, all of this ultimately traces back simply to an evolved disposition, which cannot be expected to have any more ethical reliability than any other evolved disposition, such as an egoistic one, given the ‘moral blindness’ of the forces of natural selection. The current belief in impartial utilitarianism thus traces back to a ‘contaminated starting point’ no less than current egoistic or common partialist moral beliefs (about which Singer and de Lazari-Radek remain suspicious despite the role of reasoning in refining them from the original dispositions to rational prudence and complex deontology, for example).

As before, the aspiring debunker will also claim that these ethically unreliable starting points trickled down to current belief in impartial utilitarianism with *no additional input* from recognition of *good reasons* for impartial moral concern: it's just garbage-in/garbage-out, with culture and reasoning serving only to produce more refined and consistent garbage rather than contributing genuine insight into ethical truth—just as Singer and de Lazari-Radek claim (with no stronger basis) in the case of commonsense moral beliefs. Indeed, a sceptic will claim that the very belief that *suffering is bad* or that *human good matters* is an illusion foisted upon us by evolution, explicable simply in terms of the fitness advantage to our ancestors of being disposed to believe such things, so that however much we refine such dispositions—even into Sidgwick's Universal Benevolence—we are still just refining an illusion [15]. Such a story could be told even apart from the above speculation about the evolution of initial altruistic tendencies. So the moral beliefs favoured by Singer and de Lazari-Radek are no more immune from debunkers' just-so stories than any other moral beliefs.^5^

Now the point of all this is not that there is compelling reason for us to accept any such debunking or sceptical explanatory story. I have argued that there is not, or at least that this is a complex and difficult philosophical matter and we are in any case not pushed decisively in such a direction by anything from real science. The point, however, is that if the mere possibility of such a thing were enough to debunk egoistic beliefs or commonsense beliefs in moral systems that allow for some partiality, then the belief in impartial utilitarianism prized by Singer and de Lazari-Radek would be equally vulnerable. It would be far better simply to eschew such overreaching debunking arguments, as I have advocated doing, rather than trying to make selective use of them; indeed, the considerations they cite from Sidgwick in defending against evolution-based scepticism [10] are similar to some of the points I have raised, and they can equally be used to defend against evolution-based attacks on commonsense ethical beliefs. This is not to deny the philosophical relevance of debunking *explanations* as possibilities to be aware of and to be taken seriously as challenges and potential alternatives to realist views. But as debunking *arguments*, claiming in fact to defeat our justification for some or all of our ethical beliefs, they overreach.

Even where there *is* a very plausible evolutionary influence on an ethical belief it doesn't follow that we should automatically lose confidence in that belief. Evolution has plausibly disposed us, for reasons having nothing to do with tracking moral facts, to be especially attached to our own children and to feel that we have special duties to them, or to think that suffering is bad. But it is entirely consistent with this that there are also *good reasons* for thinking it to be *true* that we have special duties to the children we have brought into the world, or that suffering *is* indeed a bad thing and that there are good reasons for mitigating it. Our ethical beliefs about these things may reflect *both* some evolutionary influences and input from recognition of good reasons for belief and action. The presence of the former, however, needn't disturb us as long as we continue, after informed and critical reflection, to find the latter credible as well—something that, again, takes us into the realm of ethical and philosophical reasoning, and cannot be settled simply from a scientific perspective.

## 5. Concluding thoughts

Recent scientific debates over such issues as the extent of the role played by kin selection in human evolutionary history, the subject of this special collection on inclusive fitness, are certainly relevant to an adequate understanding of the origins of genetically based capacities and dispositions associated with human altruism and cooperation. It is unclear, however, what bearing those debates have on cases of human thought, feeling and action that arise from the application of culturally developed, autonomous standards of ethical inquiry and deliberation, analogous to what we do in our mathematical or scientific or philosophical thinking, which is not simply controlled in its specifics by evolved dispositions of thought. I have emphasized the possibility that in drawing on more general cognitive and emotional capacities developed in cultural contexts through ethical reflection and training, we may be employing such developed capacities to discover ethical truths about how it is right to live or what we have good reason to do, much as we employ our developed and trained capacities to discover mathematical, scientific, or philosophical truths.

While there are certainly philosophical complications in developing a complete realist view along these lines, such a possibility is not precluded by anything coming from the sciences. Suppose for the sake of argument, then, that something along those lines is actually the case, as I hope at least to have made somewhat plausible through some of the examples considered. In that case, explanatory questions about why we currently think and act as we do cannot be settled without engaging philosophical and ethical issues that go beyond appeals to scientifically accessible causal factors of the sort at issue in scientific debates about the extent of kin selection among Pleistocene humans, for example. And as long as the sort of philosophical possibility in question remains a live one, we needn't, I think, be overly worried about any impending evolutionary debunking of part or all of morality or of moral realism.

## Footnotes

1 There are different forms of moral realism. According to naturalistic moral realism, there are real moral properties (such as wrongness) and facts (such as the fact that slavery is wrong), but these are exhaustively constituted by natural properties and facts. By contrast, non-naturalistic moral realism holds that moral properties and facts are (in some sense) *non*-natural, involving elements that go beyond what is usually considered to belong to the natural world. This distinction and the many complications surrounding it needn't concern us here. What matters is just that moral realism, of whatever sort, holds that there are genuinely true propositions with moral content, such as *that slavery is wrong*, and that we can know some such truths.

2 The ideas in this section draw on work developed at greater length in [3–8].

3 There are a number of complications and subtleties glossed over here that are discussed in detail in [5,6], but this summary will suffice for present purposes. See also [11] for a particularly helpful overview and critical discussion of evolutionary debunking arguments.

4 One might, of course, dispute the reliability of philosophical thought in general (especially as compared with scientific and mathematical thought), but that is irrelevant in the present context, since the debunkers themselves are obviously treating it as reliable enough to warrant taking their own philosophical arguments seriously.

5 For a related, though somewhat different, critique of the unstable use of evolutionary debunking arguments by Singer and de Lazari-Radek, see [16].

## Data accessibility

This article has no additional data.
